# Investigating the synthesis parameters of durian skin-based activated carbon and the effects of silver nanocatalysts on its recyclability in methylene blue removal

**DOI:** 10.1186/s11671-024-03974-1

**Published:** 2024-02-22

**Authors:** Dzilal Amir, Ricca Rahman Nasaruddin, Maryam Yousefi, Mohd Sufri Mastuli, Sarina Sulaiman, Md. Zahangir Alam, Nurul Sakinah Engliman

**Affiliations:** 1 Department of Chemical Engineering and Sustainability, Kulliyyah of Engineering, International Islamic University Malaysia, Jalan Gombak, 53100 Kuala Lumpur, Malaysia; 2 Bioenvironmental Engineering Research Centre, Kulliyyah of Engineering, International Islamic University Malaysia, Jalan Gombak, 53100 Kuala Lumpur, Malaysia; 3 School of Chemistry and Environment, Faculty of Applied Sciences, Universiti Teknologi MARA, 40450 Shah Alam, Selangor Malaysia; 4 Centre for Functional Materials and Nanotechnology, Institute of Science, Universiti Teknologi MARA, 40450 Shah Alam, Selangor Malaysia

**Keywords:** Activated carbon, Silver nanoparticles, Methylene blue, Durian skin, Polyvinylpyrrolidone, Citrate

## Abstract

Activated carbon (AC) is the most common and economically viable adsorbent for eliminating toxic organic pollutants, particularly dyes, from wastewater. Its widespread adoption is due to the simplicity and affordable production of AC, wherein low-cost agricultural wastes, such as durian skin can be used. Converting durian skin into AC presents a promising solution for its solid waste management. However, inherent drawbacks such as its non-selectivity, relatively short lifespan and laborious replacement and recovery processes diminish the overall efficacy of AC as an adsorbent. To address these challenges, the immobilisation of metal nanocatalysts such as silver nanoparticles (AgNPs) is one of the emerging solutions. AgNPs can facilitate the regeneration of the adsorption sites of AC by catalysing the conversion of the adsorbed dyes into harmless and simpler molecules. Nevertheless, the immobilisation of AgNPs on AC surface can be challenging as the pore size formation of AC is hard to control and the nanomaterials can easily leach out from the AC surface. Hence, in this study, we synthesised AC from durian skin (DS) and immobilised AgNPs on the AC-DS surface. Then, we used methylene blue (MB) removal for studying the adsorption capability and recyclability of the AC-DS. In the synthesis of AC-DS, the influences of reaction temperature, activating agent, and acid-washing to its capability in adsorptive removal of  MB in solution were first determined. It was found that 400 °C, KOH activating agent, and the presence of acid-washing (50% of HNO_3_) resulted in AC-DS with the highest percentage of MB removal (91.49 ± 2.86%). Then, the overall results from three recyclability experiments demonstrate that AC-DS with immobilised AgNPs exhibited higher MB removal after several cycles (up to 6 cycles) as compared to AC-DS alone, proving the benefit of AgNPs for the recyclability of AC-DS. We also found that AgNPs/Citrate@AC-DS exhibited better adsorption capability and recyclability as compared to AgNPs/PVP@AC-DS indicating significant influences of type of stabilisers in this study. This study also demonstrates that the presence of more oxygen-containing functional groups (i.e., carboxyl and hydroxyl functional groups) after acid-washing on AC-DS and in citrate molecules, has greater influence to the performance of AC-DS and AgNPs/Citrate@AC-DS in the removal of MB as compared to the influences of their BET surface area and pore structure. The findings in this study have the potential to promote and serve as a guideline for harnessing the advantages of nanomaterials, such as AgNPs, to enhance the properties of AC for environmental applications.

## Introduction

Activated carbon (AC) is regarded as a super adsorbent, which is commonly used for environmental applications such as in the removal of biological and chemical organic pollutants, as well as inorganic pollutants in water, wastewater, and air [1]. Because of its cost-effectiveness and high adsorption efficiency, AC continues to be the primary adsorbent employed in residential settings, commercial establishments, public buildings, and industrial applications. AC has excellent adsorbent properties, such as high surface area and porosity, and consists of abundant oxygen-containing functional groups such as carboxyl (–COOH) and hydroxyl (–OH) for adsorption activities. Furthermore, many low-cost agricultural wastes have been converted into AC [2–4], making it more economically feasible for industrial and consumer applications. One of them is durian skin (DS) [5, 6]. In general, durian is considered as a tropical seasonal fruit. However, due to its high demand during its peak seasons, huge waste is generated especially when the non-edible parts (the skin and seed) are about 35–50% of the whole fruit [7]. The durian wastes, especially the skin, is often disposed of in landfills without any pre-treatment, leading to leachate formation from the residual durian flesh on the skin. This leachate not only produces unpleasant odours, but also poses challenges for solid waste management. With the increasing popularity of durian and increasing efforts to make it available in year-round, there is a potential rise in the waste generation from durian. This trend is particularly noteworthy in Malaysia, where durian holds the esteemed title of "King Fruit" due to its immense popularity [7]. Therefore, converting durian wastes, especially the skin into value-added products such as AC is encouraging to solve the solid waste management of durian wastes.

Nevertheless, there are some challenges associated with AC including a restricted adsorption capacity due to absent of self-desorbed capability, especially when the adsorption sites of AC become saturated, AC can no longer effectively adsorb and remove additional contaminants. The material needs to be regenerated or replaced, which reducing its lifespan [8, 9]. The second limitation is related to regeneration of its adsorption sites which is costly [10]. In order to reuse or recycle AC, it needs to be regenerated by desorbing the adsorbed contaminants and the regeneration processes are commonly complex and require specific conditions and equipment, depending on the nature of the adsorbed substances. In some cases, it may be more practical and cost-effective to replace the spent AC with a new one. Hence, it is encouraging to investigate any methods to recycle AC and improve its lifespan. One of the reported methods for this purpose is to incorporate metal nanocatalysts which can catalyse the degradation of the adsorbed chemicals, converting them into smaller molecules which desorbed easily from the adsorption sites and later regenerate or re-expose the adsorption sites on the AC surface to the new adsorbates [11].

The advancement of nanotechnology has led to the development of numerous nanomaterials, presenting a multitude of promising environmental applications. For examples, nanomaterials such as polymer nanocomposite [12, 13], metal oxide nanoparticles [14] and noble metal nanoparticles [9, 15] have been increasingly used in environmental applications. Among all, noble metal nanoparticles have been extensively studied for the removal of organic pollutants by catalysis. For instance, silver nanoparticles (AgNPs) can function as metal nanocatalysts and bactericidal agents in the removal of hazardous chemical and biological organics pollutants in water, wastewater and air [16]. AgNPs were also reported as efficient catalysts for the catalytic removal of dyes in wastewater [17–19]. However, in a free suspension form, AgNPs are hard to be recycled and reused again, hence, many studies had to immobilise AgNPs on support materials such as metal oxides, polymers, and AC [20]. Furthermore, in a free suspension form, AgNPs can easily aggregate into larger particles, hence impeding their catalytic activity. However, immobilisation of metal nanoparticles like AgNPs on AC suffers from several limitations. As the pore size of AC is not consistent and hard to control, the attachment of AgNPs on AC surface may not be strong and they can easily leach out during the catalytic reaction, adsorption process, and washing for recyclability in the removal of water and wastewater pollutants.

In the environmental applications, AC remains as the most popular adsorbent for practical removal of organic pollutants in wastewater. Dyes such as methylene blue (MB) are among the common organic pollutants discharged mainly from textile industry [21] and being removed extensively by AC [10]. This organic pollutant is chemically stable and have little biodegradability [22]. It may cause disorder to the aquatic system by inhibiting sunlight from getting to the water and causes reduction in photosynthesis [1, 23]. Although MB is not considered to be a very toxic dye, it can still affect human health as its continuous exposure can cause hypertension, mental confusion, methemoglobinemia, vomiting, skin staining, anaemia, and nausea, precordial pain, and excessive sweating [15, 24]. As the removal of dye like MB is encouraging for wastewater treatment, we chose MB as our model organic pollutant. In addition, the visible blue colour of MB contributes to its widely application as model dye pollutant for laboratory studies in evaluating the adsorption capacity of AC and catalytic activity of AgNPs. The bright blueish MB solution turns into light blue and toward colourless solution after a complete adsorption of MB on AC and after its degradation into smaller molecules catalysed by AgNPs. The degradation of MB into smaller molecules commonly occurs by oxidation reaction with the addition of oxidants such as hydrogen peroxide. Both adsorption and degradation of MB can quantitatively be evaluated by using a UV–Vis spectrophotometer [25].

This study demonstrates the influences of synthesis conditions of AC from DS, immobilisation of AgNPs on AC-DS, as well as evaluating the adsorption and recyclability of the AC-DS, with and without the immobilised AgNPs, in the removal of MB in solution. The studied parameters in the AC-DS synthesis were calcination temperature, activating agent, and acid-washing. Meanwhile, AgNPs used in this study were synthesised in-house using two types of stabilisers, which were anionic molecules (citrate) and polymer (polyvinylpyrrolidone, PVP). The effects of these stabilisers to the morphology of the AgNPs, immobilisation of AgNPs on AC-DS, as well as the adsorption and recyclability of the AC-DS immobilised with AgNPs were also studied. The novelty of this study is mainly from the combination of AC synthesised from durian wastes and with the incorporation of AgNPs to improve the adsorption capability and recyclability of the AC-DS. This study also demonstrates the effects of two types of stabilisers which are citrate and PVP to the synthesis of AgNPs, immobilisation of the AgNPs onto AC-DS surface, and the adsorption capability and recyclability of the AC-DS. To best of our knowledge, there is still insufficient studies on improving the properties of AC, which was synthesised from durian wastes, especially by adding nanomaterials. In addition, in terms of nanomaterials understanding, we also realize that there is insufficient comparative study to evaluate the effects of stabilisers to the metal nanoparticles-AC nanocomposites for their environmental application. Hence, we assert that this study makes noteworthy contributions, paving the way for additional research endeavours exploring the application of nanomaterials in environmental contexts.

## Materials and methods

### Materials

Ultrapure laboratory-grade Millipore water (18.2 MΩ) was used in the synthesis of AgNPs while laboratory-grade deionized water was used in the synthesis of AC, immobilisation of AgNPs onto AC surface, and MB removal experiments. Silver nitrate (AgNO_3_) in form of ACS reagent powder (with ≥ 99% purity), polyvinylpyrrolidone (PVP) in powder form (with average molecular weight of 7,000), trisodium citrate dihydrates, nitric acid (HNO_3_) in form of ACS reagent (with 70% v/v concentration) and sodium borohydride (NaBH_4_) in powder from (with ≥ 96% purity) were purchased from Sigma-Aldrich–Merck. Other chemicals, such as potassium hydroxide (KOH) and sodium hydroxide (NaOH) flakes (with 98% purity and soap production grade) were purchased from a local supplier (Chemistree) through the local online market platform (Lazada). Meanwhile, hydrogen peroxide (H_2_O_2_) solutions (with 20% v/v and 50% v/v concentration and laundry and detergent grade) were purchased from a local supplier (In-Scent) through the local online market platform (Shopee). Finally, methylene blue (MB) in powder form (with 100% purity and Bendosen brand) was purchased from a local supplier (A&D World Enterprise), also through the local online market platform (Shopee).

### AgNPs Synthesis and characterisations

The synthesis of AgNPs was done according to method established in our previous study [26]. Briefly, silver precursor, AgNO_3_ solution (0.05 mL; 40 mM) was mixed with ultrapure water in a 20 mL glass vial. Then, stabiliser solution (PVP or citrate) was added at room temperature (27 °C). The concentration of the stabiliser was 40 mM (similar to AgNO_3_) while the volume of the stabiliser was tailored based on the variation in the molar ratio of AgNO_3_-to-Stabiliser (R_Ag/S_). The ultrapure water was again added to the mixture to make-up the total volume of 3 mL. The mixture was hand shaking for 10 s and allowed to mix for 10 min. After 10 min, NaBH_4_ solution (0.02 mL; prepared by dissolving 40 mg of NaBH_4_ powder in 10 mL of ultrapure water) was added into the solution dropwise and the mixture was allowed to etch without any mixing at room temperature. The color of the solution turned yellowish at the beginning of the reaction and the yellow color became stronger (brownish yellow) over time, indicating the formation of AgNPs. After 70 min, the solution was analyzed by UV–Vis spectrophotometer (JASCO, V730) under spectrum mode with the wavelength ranging from 300 to 700 nm. Notably, the color of AgNPs can be too strong. Due to the limitation of the UV–Vis spectrophotometer, the sample was diluted by mixing as-synthesised AgNPs solution (0.2 mL) with ultrapure water (1.8 mL) in a 3 mL quartz cuvette. The UV–Vis absorption was recorded. Selected as-synthesised AgNPs were also characterised by TEM (Zeiss Libra 120) at iCRIM, University Kebangsaan Malaysia, Bangi, Malaysia. All experiments were done in triplicates. The concentration and volume of all reagents are summarized in Table 1.

**Table 1:** Variations in volume of silver precursor and stabiliser in the synthesis of AgNPs

Molar ratio of AgNO_3_ -to-stabiliser (R_Ag/S_) (PVP or citrate)	40 mM AgNO_3_ (mL)	40 mM Stabiliser (mL)	Ultrapure water (mL)
1:1	0.05	0.05	2.9
1:3	0.05	0.15	2.8
1:5	0.05	0.25	2.7
1:7	0.05	0.35	2.6

### Synthesis and characterisation of AC

**Fig. 1:**
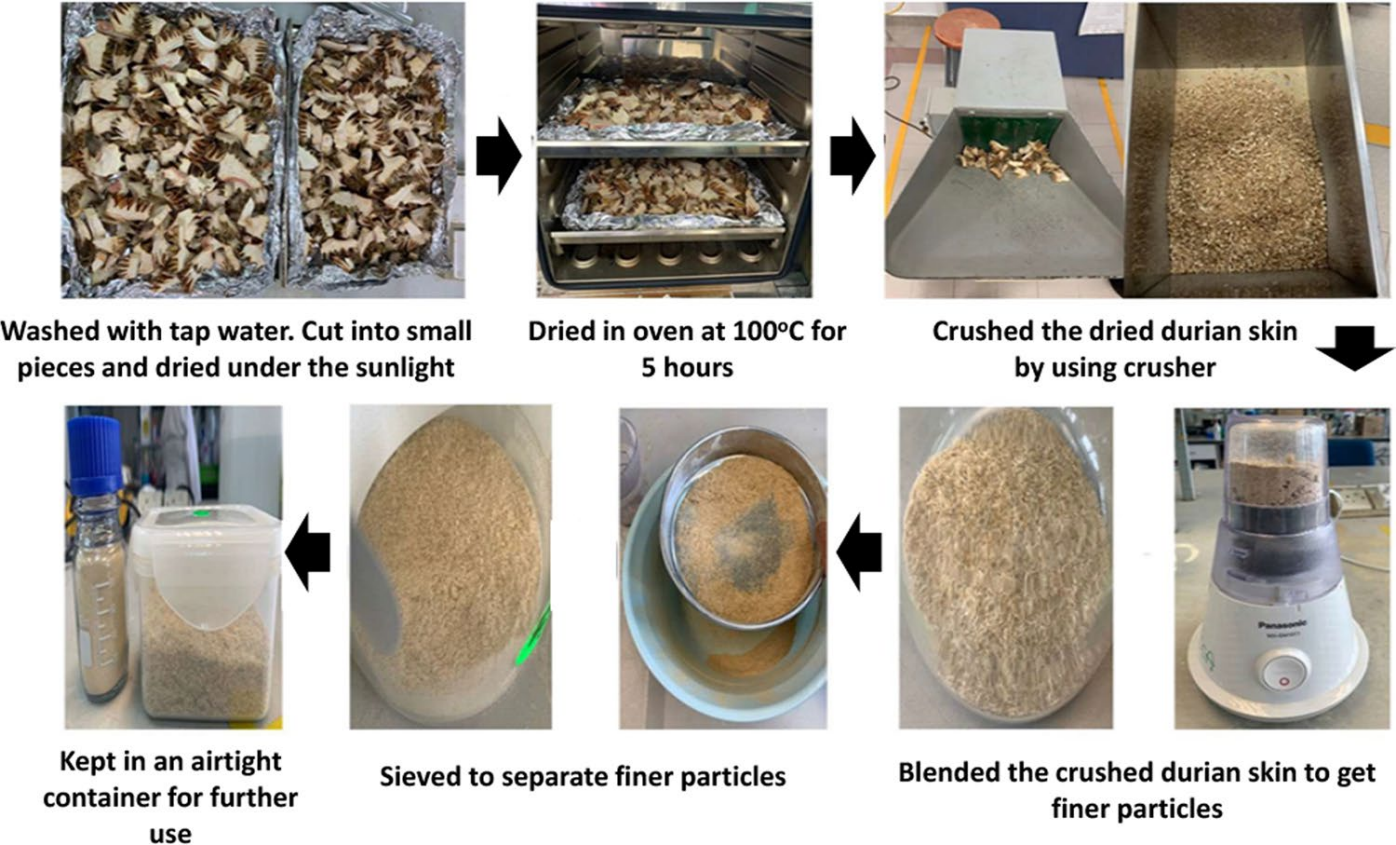
Preparation of the durian skins powder

Before being used, the DS was pretreated as shown in Fig. 1. Briefly, DS was collected from a local durian seller. Initially, DS was cleaned with normal tap water to eliminate the remaining durian flesh. Then, they were cut into smaller sizes and dried under the sun for a few hours. The drying process was continued in oven at 100 °C for 5 h to further remove the moisture content and any remaining flesh on the DS. Then, the dry DS was crushed, grinded and sieved to get a finer or powder-like structure as in Fig. 1. Finally, this pretreated DS powder was kept in an airtight container for further use.

Meanwhile, for the synthesis of AC, there are several names used for the thermal process such as pyrolysis [22], calcination [26], and carbonization [27]. Pyrolysis and carbonization processes occur in the absence of oxygen or with very limited oxygen, while calcination may involve the presence of little air and oxygen. Pyrolysis and carbonization primarily produce a carbonaceous residue, while calcination may lead to the formation of oxides and unwanted ashes. In this study, the synthesis of AC-DS was carried out through the calcination method in an enclosed porcelain crucible and in ashing furnace, according to reported study by Yuliusman et al. [28] with slight modifications. This process took place in an environment with restricted air and oxygen, ensuring controlled conditions during calcination. The use of crucible caps played a pivotal role in minimizing the presence of oxygen, thereby mitigating the risk of excessive ashes formation, and fostering the generation of AC.

Briefly, the DS powder was weighed up to 1 g and transferred into a 30 mL crucible. Then 1 g of activating agent was added with mass ratio of durian skin-to-activating agent of 1:1. Subsequently, 5 mL of deionized water was added, and the mixture was stirred using a spatula to make them homogenous and the mixture was allowed to react for 30 min at room temperature (27 °C). After that, the crucible was closed and the mixture of DS powder, activating agent and water was calcined for 1 h at selected temperature inside a 3 L ashing and burn-off chamber furnace (Carbolite). After 1 h, the calcined product was transferred into a 15 mL falcon tube for washing. In the washing step, the as-synthesised AC-DS in the falcon tube was mixed with deionized water, centrifuged, and the supernatant was discarded until the supernatant had a neutral pH (pH 7 ± 0.5). Some samples underwent acid-washing with HNO_3_ (50% v/v concentration) once before being washed with deionized water. It was approximately 10 washing cycles needed to reach the neutral pH for samples with and without acid-washing. The AC-DS was then dried in the oven (60 °C) overnight to remove the remaining water and moisture content. The characterisations of the AC-DS was done by FTIR (Bruker, Invenio-S), SEM–EDS (JEOL, JSM 5610) and XRD (Bruker, D2 Phaser) machines. All experiments were done in triplicates. Gas adsorption test for characterising the surface area and pores of all synthesised AC-DS were also done using BELSORP-mini II instrument from BEL Japan Inc. and nitrogen gas (N_2_) as adsorptive. The N_2_ adsorption–desorption of the AC-DS were measured at − 196 °C. Before the measurement, the sample was degassed at 200 °C for 6 h in a vacuum oven. The isotherm was further analysed using the BET (Brunauer–Emmett–Teller) and BJH (Barrett-Joyner-Halenda) methods to give the specific surface area, total pore volume and average pore diameter of the AC-DS.

In the synthesis of AC-DS, we conducted a comparative analysis of the reaction conditions by systematically varying parameters. Firstly, we varied the calcination temperature. Secondly, we varied the activating agent and finally, we varied the acid-washing. The variations of the reaction conditions at different stages are summarized in Table 2.

**Table 2:** Variations of parameters in the synthesis of AC-DS

Stage/parameters	Stage 1: Temperature	Stage 2: Activating agent	Stage 3: Acid-washing
Variation in the parameter	400 °C versus 600 °C	KOH versus NaOH	With versus without acid-washing (HNO_3_ with 50% v/v concentration)
Other consistent parameters	Activating agent (KOH) and washing with deionized water only	Calcination temperature (400 °C) and washing with deionized water only	Activating agent (KOH) and calcination temperature (400 °C)

### Adsorptive removal of MB by AC-DS

The reaction conditions in the synthesis of AC-DS, selected for further study were determined by the MB removal through adsorption capability of AC-DS. Briefly, MB solution (0.2 g/L) was prepared using deionized water. Then, 10 mg of AC-DS was weighed using the 0.001 g high-precision digital gold weighing scale and then it was transferred into a 3 mL cuvette. After that, MB solution (1.5 mL; 0.2 g/L) was added to the cuvette slowly. A control experiment without the presence of AC-DS was also prepared. The adsorption process was allowed to happen by leaving all the cuvettes including the control inside a closed box, at room temperature and without any mixing for 20 h. MB solution peak was measured by UV–Vis spectrophotometer (JASCO, V-730) after 20 h. Most of the AC-DS were settled down at the bottom of the cuvette due to their heavier size and gravity effect, thus centrifugation was not needed. However, some of the AC-DS was found floating in the MB solution, indicating that the floating AC-DS did not have sufficient hydroxyl or carboxyl functional groups. The graph for each spectrum was plotted. The value of UV–Vis absorbance at wavelength 662 nm was taken for the calculation of the percentage of MB removal (%) by adsorption. All experiments were done in triplicates. The equation for the percentage of MB removal (%) is as follow:

1 $$Percentage\;of\; MB\;removal\;\left( \% \right) = \frac{{\left( {Abs_{C} - Abs_{S} } \right)}}{{Abs_{C} }} \times 100\%$$

whereby ${Abs}_{C}$ is the UV–Vis absorbance at 662 nm of MB solution in the control experiment and ${Abs}_{S}$ is the UV–Vis absorbance at 662 nm of MB solution for sample after the adsorption or catalytic reaction for AC-DS immobilised with AgNPs. Whenever needed, the plots were normalized to ensure fair comparison of all points in the graphs can be made, and the value of UV–Vis absorbance at 662 nm was obtained from the normalized data.

### Immobilisation of AgNPs on AC-DS

AgNPs stabilised by PVP (AgNPs/PVP), and citrate (AgNPs/Citrate) were then immobilised on AC-DS. The concentration of the AgNPs solution was 0.67 mM based on moles of Ag. Based on the results of AgNPs synthesis reported in our previous study [26], the optimum condition in terms of molar ratio, R_Ag/S_ were 1:1 and 1:3 for AgNPs/PVP and AgNPs/Citrate, respectively. However, the sample of AgNPs/PVP synthesised with R_Ag/S_ of 1:1 was not stable after 1.5 years. They were aggregated into blackish precipitates at the bottom of the vial. Therefore, AgNPs/PVP solution synthesised with R_Ag/S_ of 1:3 which was stable after 1.5 years was selected for further study. Similarly, AgNPs/Citrate synthesised with R_Ag/S_ of 1:3 was also selected for further study. The immobilisation of both AgNPs was done by deposition–precipitation method [29]. Briefly, AC-DS was weighed up to 10 mg using the 0.001 g high-precision digital gold weighing scale and was transferred into a 2 mL microcentrifuge tube. Then, 0.1 mL of AgNPs/PVP or AgNPs/Citrate solution was added into the AC-DS in the tube, followed by the addition of 0.4 mL of deionized water. The mixture was mixed by hand shaking. AC-DS immobilised with the AgNPs was allowed to precipitate at the bottom of the tube by centrifugation at 7000 rpm for 5 min. Then, the supernatant (remaining liquid) was carefully pipetted out from the tube and the immobilised AC-DS with AgNPs was dried at room temperature. When the brownish colour of AgNPs solution becomes colourless or less brownish, this indicate the AgNPs in the solution were already adsorbed and immobilised on the AC-DS surface. All experiments were done in triplicates. The dry AgNPs/Citrate@AC-DS and AgNPs/PVP@AC-DS were further characterised by FTIR (Bruker, Invenio-S), SEM–EDS (JEOL, JSM 5610) and XRD (Bruker, D2 Phaser) machines. Gas adsorption test for characterising the surface area and pores of AgNPs/Citrate@AC-DS and AgNPs/PVP@AC-DS were also done using using BELSORP-mini II instrument from BEL Japan Inc. and nitrogen gas (N_2_) as adsorptive. The N_2_ adsorption–desorption of the AC-DS were measured at − 196 °C. Before the measurement, the sample was degassed at 200 °C for 6 h in a vacuum oven. The isotherm was further analysed using the BET and BJH methods to give the specific surface area, total pore volume and average pore diameter of the samples. The results were compared with surface area and pore characteristics of AC-DS alone.

### Recyclability test using MB removal through adsorption and catalytic oxidation

The model reaction used for assessing the recyclability and catalytic activity of the AC-DS and AC-DS immobilised with AgNPs was MB oxidation using hydrogen peroxide (H_2_O_2_) solution (with 20% v/v concentration) as the oxidizing agent. In this oxidation reaction, MB molecule was degraded into smaller molecules such as H_2_O and CO_2_ which can easily desorbed from the AC adsorption sites. The reaction was done inside a 2 mL microcentrifuge tube. For the first cycle, each sample (AC-DS and AC-DS immobilised with AgNPs) was weighed up to 10 mg using the 0.001 g high precision digital gold weighing scale and was transferred into the microcentrifuge tube. Then, in a separate tube, a mixture of MB solution (0.5 mL; 0.125 g/L) and H_2_O_2_ solution (0.5 mL) was prepared. The mixture was then added into the earlier microcentrifuge tube containing the sample. The tube was hand shaking for 10 s and then, reaction was allowed to happen overnight for 2 days to ensure all MB were adsorbed by all samples in the first cycle. It was expected that some adsorbed MB on AC-DS immobilised with AgNPs were catalysed by the AgNPs, thus regenerate the adsorption sites on the AC-DS for the second cycle. After 2 days, the solid and liquid in the tube were separated using filter paper. The UV–Vis absorption of the filtrate was measured using UV–Vis spectrophotometer (JASCO, V-730).

Two control experiments (MB with deionized water and MB with H_2_O_2_ solution only) were also prepared and passed through the filter paper. Some of the MB was adsorbed and absorbed to the filter paper. The remaining MB in the filtrate was measured through UV–Vis spectrophotometer. The filtrate for the first control which was the mixture of MB with deionized water was used to determine the ${Abs}_{C}$ for the calculation of percentage of MB removal (%) as shown in Eq. 1. Then, the second cycle of reaction was done by putting the samples in a 10 mL vials and then, a mixture of MB solution (1 mL; 0.125 g/L) and H_2_O_2_ solution (1 mL) was added into the vial, followed by the addition of 2 mL of deionized water. The vial was mixed by hand shaking for 10 min and the reaction was allowed to happen for an hour. The reaction time in the second cycle was made shorter than the first cycle to ensure the comparisons among AC-DS, AgNPs/PVP@AC-DS and AgNPs/Citrate@AC-DS can be obtained. The filtrate was obtained by filtration using filter paper. Similarly, two control experiments (MB with deionized water and MB with H_2_O_2_ solution only) were also prepared and passed through the filter paper. Some of the MB was adsorbed and absorbed to the filter paper. The remaining MB in the filtrate was measured through UV–Vis spectrophotometer. The filtrate for the first control (MB with deionized water only) was used to determine the ${Abs}_{C}$ for the calculation of the percentage of MB removal (%) as shown in Eq. 1. All experiments were done in triplicates.

The recyclability test was repeated with slight modifications to its experimental method. In the repeated recyclability test, UV–Vis absorption was measured using UviLine 9400 spectrophotometer by Secomam due to the malfunction of UV–Vis spectrophotometer (JASCO, V-730) that was used previously. The selected wavelength with the highest peak of the MB solution was 658 nm as compared to 662 nm when using previous UV-Vis spectrophotometer (JASCO, V-730) . No filter paper was used this time to prevent the throwing of more fine and smaller particles of AC when transferring the liquid out from solid samples (AC-DS, with and without immobilised AgNPs). In the first cycle, 10 mg of sample (AC-DS, AgNPs/Citrate@AC-DS or AgNPs/PVP@AC-DS) was mixed with 0.5 mL of MB solution (0.125 g/L) and 0.5 mL of H_2_O_2_ solution (20%) in a 2 mL microcentrifuge tube. After 20 h, the mixture was centrifuged for 20 min at 10,000 rpm. Only 700 mL supernatant was collected by 100 mL pipette carefully and transferred into a 3 mL cuvette for UV–Vis absorption analysis. Remaining 0.3 mL liquid was left with the sample. This step is important to avoid significant reduction of sample, especially AC with very fine and small size particles. Two control experiment were also prepared which the first control had 0.5 mL of MB solution (0.125 g/L) and deionized water only and another control had 0.5 mL of MB solution (0.125 g/L) and 0.5 mL of H_2_O_2_ solution (20%). Total volume of liquid in the first cycle was 1 mL. For the second cycle, a new 0.5 mL of MB solution (0.125 g/L) and 0.5 mL of H_2_O_2_ solution (20%) were added into the same 2 mL microcentrifuge containing the remaining sample and liquid. New control experiments were also prepared with the additional of 0.3 mL of deionized water, to ensure total volume of liquid for all samples including controls was about 1.3 mL. The mixture was mixed by hand shaking for 10 s and then centrifuged for 20 min at 10,000 rpm. 1 mL of the supernatant was collected, and its UV–Vis absorption was measured. Next, for the third, fourth, fifth and the sixth cycles, the procedure of second cycle was applied.

### Leaching of immobilised AgNPs

Simple experiment for testing the leaching of immobilised AgNPs from the AC-DS was also performed. Due to the absent and limited access of Inductively coupled plasma—optical emission spectrometry (ICP-OES), the quantification of element (silver) was done using SEM–EDS (JEOL, JSM 5610). The weight and atomic percentage provide indirect evidence on the presence of AgNPs in the supernatant due to their leaching from the AC-DS. The experiment was done together with the recyclability test. The remaining supernatant after the first cycle, was transferred into 20 mL vials and then one round filter paper, cut into 5 mm diameter was added into the vial. The vial was dried overnight in the oven at 60 ± 5 °C overnight. Then, the dry filter paper was analysed under SEM–EDS without the addition of any conductive metal coating. The analysis was done with controlled operating conditions (20 kV, 200 × magnification, 10 mm working distance, 30 times scan for the elemental analysis and only silver and oxygen were included in the quantification of the elements). Three control samples which were filter papers wetted and dried with AgNPs/Citrate and AgNPs/PVP solutions (prepared by mixing 0.1 mL of the catalysts solution and 0.4 mL deionized water) and supernatant from MB removal test using AC-DS only were also included in the SEM–EDS analysis for a proper comparison. Although this method is simple and provide indirect evidence for the silver leaching, this method is considerably safer for analysing silver content because the presence of fine AC particles in the supernatant, might damage the ICP-OES machine.

## Results and discussion

### Silver nanoparticles (AgNPs)

The effects of synthesis conditions such as the molar ratio of stabiliser-to-AgNO_3_ (R_Ag/S_) and in the synthesis of AgNPs can be referred to our published previous study [26]. After 1.5 years, the AgNP solutions which were stored in the fridge at − 4 °C were observed before being used for immobilisation. It was found that the AgNPs/PVP synthesised with R_Ag/S_ = 1:1 was unstable and already aggregated into blackish precipitates at the bottom of the vial. Therefore, AgNPs/PVP and AgNPs/Citrate solutions synthesised with R_Ag/S_ = 1:3 were selected for the immobilisation study because they were still stable as AgNPs. These selected AgNPs were further characterised using TEM.

**Fig. 2:**
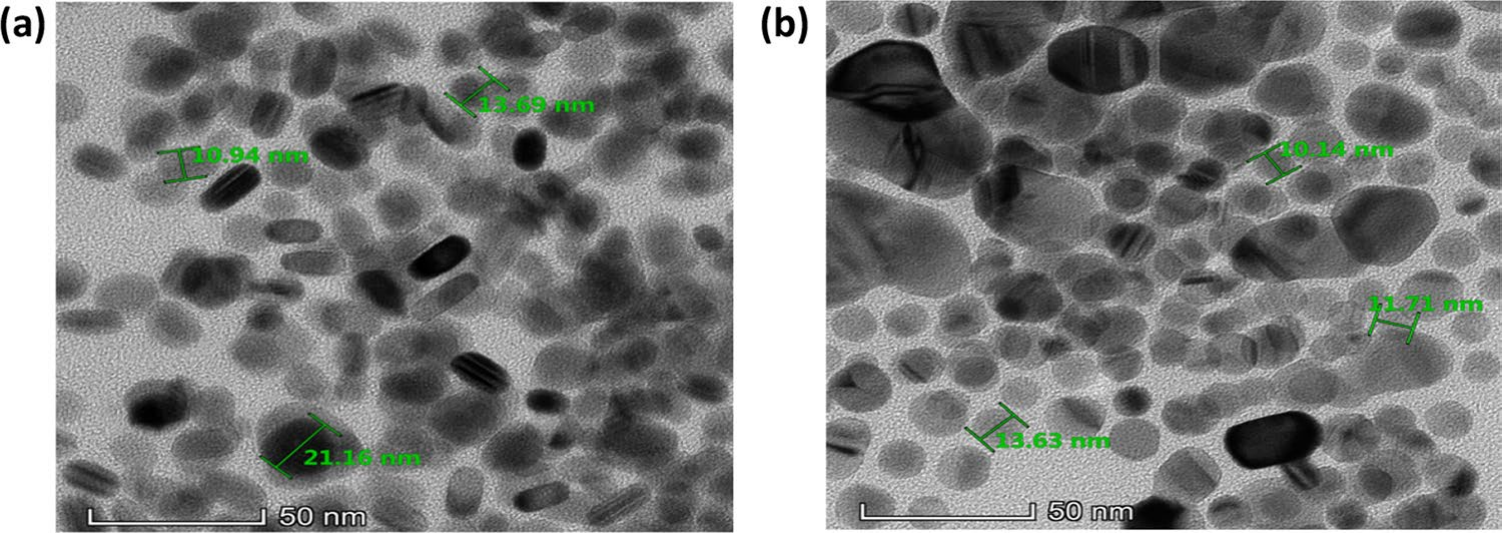
TEM image of as-synthesised **a** AgNPs/PVP and **b** AgNPs/Citrate solutions after 1.5 years kept in the freezer at −4 °C. Both AgNPs were synthesised with R_Ag/S_ of 1:3

As shown in Fig. 2a, b, AgNPs/PVP and AgNPs/Citrate were polydisperse in size and shape. Based on the TEM image in Fig. 2a, AgNPs/PVP had both spherical and rod-like shapes. PVP molecules can adsorb onto the growing crystal faces of the nanoparticles, acting as a template or capping agent that directs the anisotropic growth along specific crystallographic directions. This preferential growth leads to the formation of rod-shaped nanoparticles [30]. In other reported study, it was found that majority of AgNPs synthesised using PVP as stabilisers produced spherical shape [31]. Probably, the rod-shaped AgNPs/PVP in this study were formed after the samples was kept for a long time, which was 1.5 years. Meanwhile, in terms of shape, AgNPs/Citrate were less polydisperse as most of the nanoparticles had spherical shape. Citrate is known for its ability to reduce and stabilise AgNPs, but it generally favours the formation of round or spherical nanoparticles [32]. Citrate molecules can interact with the silver ions in the precursor solution, forming a protective layer around the nanoparticles during their synthesis. The spherical shape is a result of isotropic growth, where crystal growth occurs equally in all directions [32]. Other than affecting the shape of the AgNPs, the choice of stabiliser can also influence the size of AgNPs significantly. Majority of AgNPs/PVP had size ranging from 10 to 20 nm. Meanwhile, AgNPs/Citrate favour spherical nanoparticles similar to previous reported study [33]. Majority of AgNPs/Citrate had size ranging from 10 to 15 nm. In term of size, AgNPs/PVP had better dispersion as compared to AgNPs/Citrate as we could observe few big nanoparticles with size ranging from 20 to 50 nm in the AgNPs/Citrate sample.

### Effects of reaction conditions in the synthesis of AC-DS

**Fig. 3:**
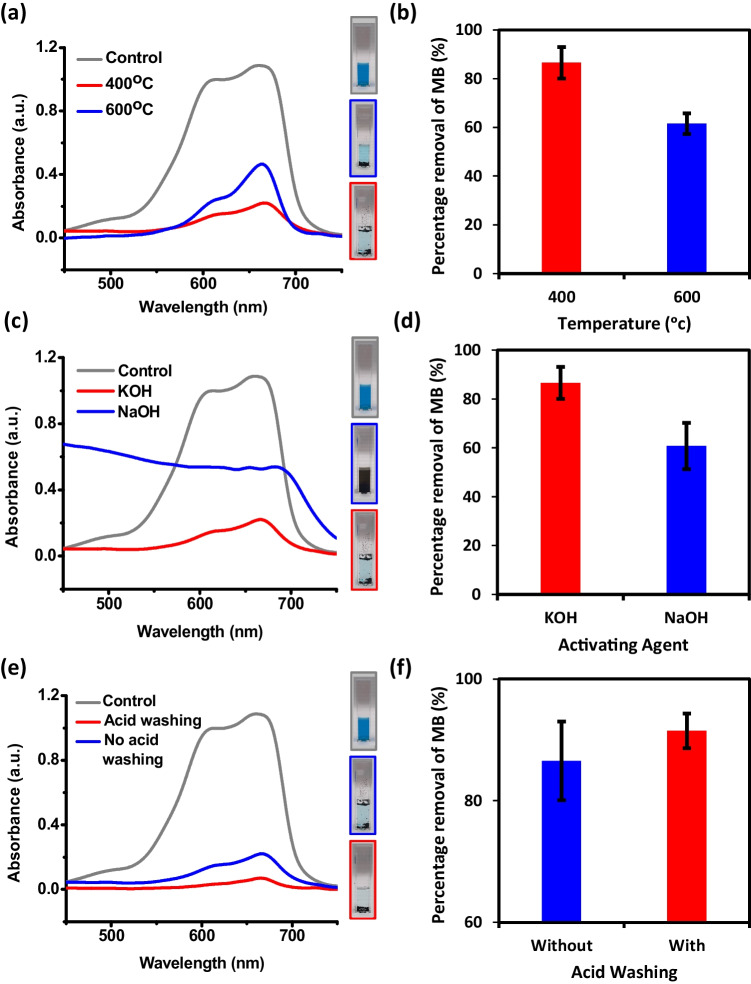
Effects of **a**, **b** calcination temperature, **c**, **d** activating agent, and **e**, **f** acid-washing on the adsorptive removal of MB in solution. Results in **a**, **c**, and **e** show UV–Vis absorption spectra of MB solutions while results in **b**, **d**, and **e** show the percentage of MB removal (%). Images inside each line graph show the colour of solutions after the adsorption test

As mentioned in the methodology section, the synthesis of AC-DS was conducted using calcination process with limited amount of air and oxygen using chemical activation method. The activating agent (KOH or NaOH) and acid-washing (with HNO_3_) were used to create pores and introduce hydroxyl and carboxyl functional groups on the surface of AC-DS. Meanwhile, calcination temperature is the most influential parameter for the formation of AC-DS. High temperature is required to make more pores structure, thus increasing the surface area-to-volume ratio of the AC-DS. We systematically varied and upheld specific parameters across different stages of the experiment. The best operating conditions were determined mainly from the adsorption capacity of the AC-DS by performing adsorptive removal of MB in solution. Results for the influences of reaction conditions in the synthesis of AC-DS are demonstrated in Fig. 3.

We initiated our investigation of reaction conditions in the AC-DS synthesis by comparing the influence of calcination temperature, recognizing its pivotal role in shaping the quality, formation of pores, yield, and energy consumption associated with AC synthesis. It was found from the MB removal test in Fig. 3a that AC-DS calcined at 400 °C shows a clearer solution as compared to AC-DS calcined at 600 °C, showing higher MB removal by adsorption. Based on the results in Fig. 3b, the percentage of MB removal when using AC-DS calcined at 400 °C is higher (86.55 ± 6.48%) than AC-DS calcined at 600 °C (61.56 ± 4.27%). In addition, elevated temperatures were found to contribute to an increased formation of ashes, consequently reducing the overall yield of AC-DS post-washing. The heightened pressure within the crucible at higher temperatures led to the inadvertent opening of the crucible cap during calcination. This exposure to air facilitated the raw materials reaction with oxygen, promoting the formation of additional ashes in the process. In addition, the operating cost can be reduced by using low temperature (400 °C) as compared to high temperature (600 °C).

Our second investigation focused on the impact of the activating agent (NaOH and KOH), revealing a noteworthy finding in Fig. 3c–d. In terms of colour changes, the MB solution for AC-DS activated by KOH was clearer compared to that of NaOH, indicating more MB being adsorbed by the AC-DS activated by KOH. The use of KOH resulted in the breakage of longer fibers and exfoliation from biomass DS which improved the porosity of the AC-DS. The activation with KOH can also be attributed to the potassium interaction and the stretching of carbon layers [34]. We also observed a higher formation of ashes for AC-DS synthesised with NaOH as the activating agent. This phenomenon can be attributed to the lower dissociation energy of NaOH (77 ± 4 kcal/mol) as compared to KOH (81 ± 2 kcal/mol) [35]. In aqueous environments, NaOH readily and easier to dissociates into Na^+^ and OH^−^ ions as compared to KOH. The presence of these OH^−^ ions acted as oxidants during calcination, contributing to the heightened formation of ashes in the process, hence reducing the yield of AC-DS.

In addition, KOH mechanism in the formation of the pores is complex and contains the disintegration (almost explosively) of the structure following intercalation and some gasification by oxygen molecules of hydroxide as well [36]. Many mesopores with an irregular yet extremely porous structure were occupied by the AC-DS synthesised using KOH as an activating agent. Bardhan et al. [37] also found that the MB adsorption on AC-DS, activated by KOH was greater than AC-DS activated by NaOH because the pores in the KOH micrograph were greater than in the NaOH micrographs. Based on the bar graph in Fig. 3d, the percentage of MB removal when using NaOH was only 60.77 ± 9.48%, lower than the percentage of MB removal by AC-DS synthesised using KOH as activating agent (86.55 ± 6.48%), indicating that AC-DS activated by KOH had better adsorption capacity as compared to AC-DS activated by NaOH.

In our final investigation pertaining to acid-washing, we observed a positive impact on adsorption of AC-DS after washing it with acid. The addition of acid-washing proved beneficial by introducing extra carboxyl and hydroxyl functional groups to the AC-DS surface [38]. These functional groups enhance the hydrophilicity of the AC-DS, facilitating better mixing with MB solution. The improved hydrophilicity contributes to enhanced adsorption properties, emphasizing the role of acid-washing in modifying the surface chemistry of AC-DS for improved interaction with target substances in aqueous environments. In Fig. 3e–f, it was found that the AC-DS with acid-washing resulted in a clearer MB solution after 24 h as compared to that of AC-DS without acid-washing. This indicates that acid-washing did improve the adsorption of AC-DS (91.49 ± 2.86% MB removal) as compared to AC-DS without acid-washing (86.55 ± 6.48% MB removal). As mentioned previously, acid-washing is preferable to enhance the removal of impurities and ashes content, enhance pore enlargement, and introduce extra functional groups onto AC-DS. Hence based on the effect of calcination temperature, activating agent and acid-washing, AC-DS synthesised using KOH at 400 °C and with acid-washing was selected for further study.

### Characterisations of AC-DS

**Fig. 4:**
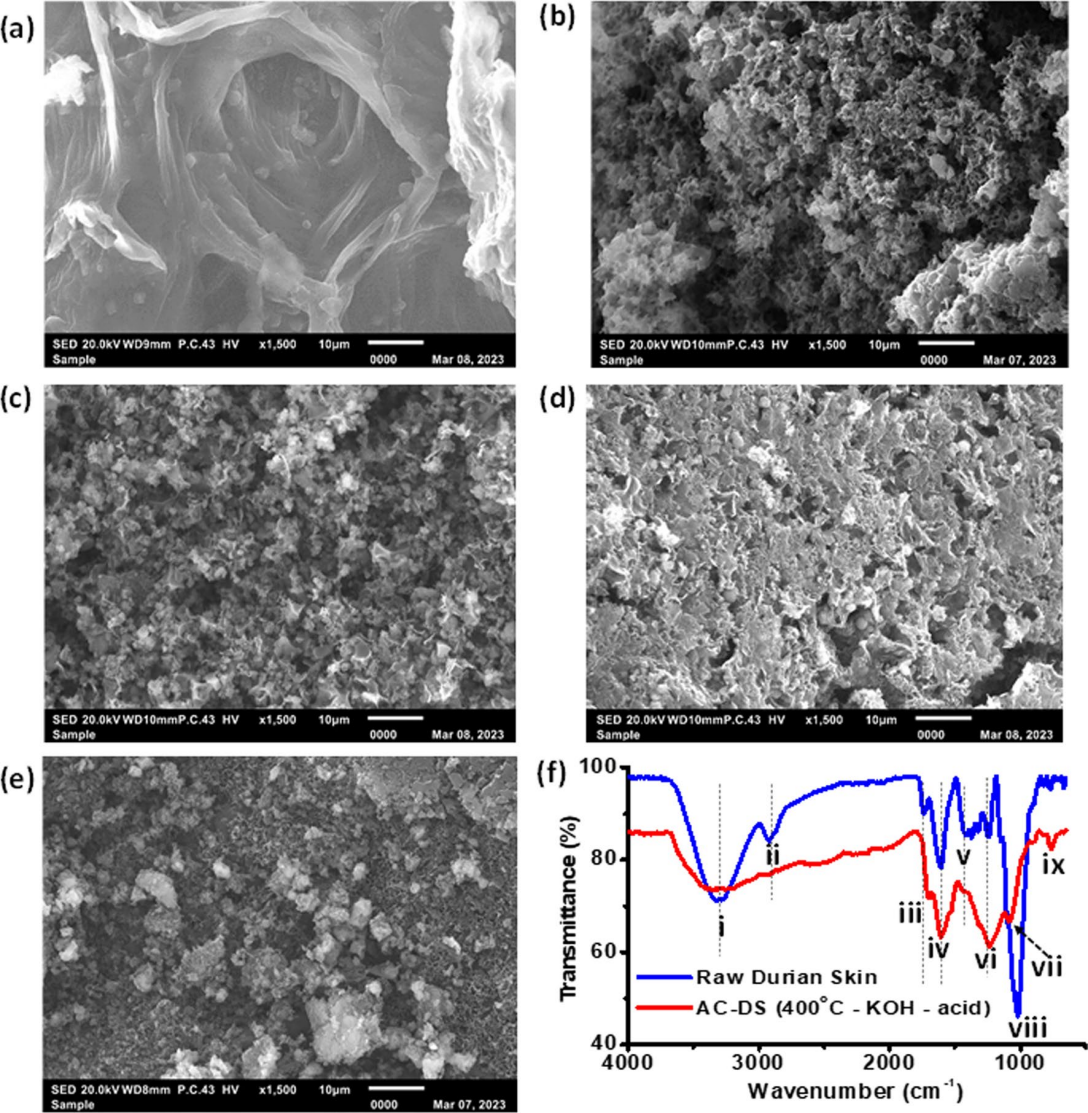
SEM images with 1500 × magnification of **a** raw DS powder, **b** AC-DS synthesised at 400 °C with KOH activating agent and without acid-washing, **c** AC-DS synthesised at 600 °C with KOH activating agent and without acid-washing, **d** AC-DS synthesised at 400 °C with NaOH activating agent and without acid-washing and **e** AC-DS synthesised at 400 °C with KOH activating agent and with acid-washing. **f** FTIR graphs for raw DS powder and AC-DS synthesised at 400 °C with KOH activating agent and with acid-washing

Figure 4 shows the SEM images for AC-DS activated using different calcination temperature, activating agents and acid-washing. The SEM analysis reveals that the raw durian skin did not have any pores structures. However, the treated AC-DS with different activating agents gave more porous structures with honeycomb-like shapes with different pore sizes owing to the degradation of the cellulosic fraction, which was affected by the thermal decomposition of biomass [39]. The porosity of AC-DS calcined at 400 °C was much smaller due to the lower temperature applied during calcination with a controlled oxygen supply hence reducing energy to open of porosity. Lower temperature was considered the optimum condition in this study; besides eliminating impurities and volatile substances, it also preserves some original properties of AC which is useful in adsorption reaction for the next experiment and reduces the cost of operation as compared to higher calcination temperature. Meanwhile, according to Su et al. [40], acid-washing enhances the removal of impurities and ashes content, pore enlargement, and introduces more functional groups onto the AC surface, hence improving its adsorption properties. Finally, in terms of activating agents, KOH tends to create AC with larger pore volume which can accommodate larger and increase the material’s adsorption capacity. Between AC-DS activated by KOH and AC-DS activated by NaOH, AC-DS activated by KOH at 400 °C and with the presence of acid-washing (labelled as 400 °C-KOH-acid) was selected for further study.

The details of FTIR peaks in Fig. 4f are summarised in Table 3. The FTIR results show the surface functional groups of raw durian skin with the selected AC-DS (400 °C-KOH-acid). Both raw durian skin and AC-DS (400 °C-KOH-acid) contains O–H stretch for phenol and hydroxyl (strong and broad peak) and N–H stretch for amide and 1″ and 2″ amine (medium peak) in **region i**. However, the peak is more intense before calcination process due to breakdown of functional group after calcination heat was applied. In **region viii**, there is S=O stretch for sulfoxide (strong peak) as DS contains sulphur-containing compounds which contribute to strong pungent odour. This region could also indicate the presence of carbon–carbon ring stretching or carbon–nitrogen stretching which presence in the biomass based on the sharp FTIR peak at 1012 cm^−1^ (close to the **peak viii** in this study) which was observed for biomass samples in an existing study reported by Bilal et al. [41]. However, after calcination, the sulfoxide functional group was eliminated and only C–O stretch for carboxyl and hydroxyl (variable peaks) were remained, indicating the oxidation and removal of sulphur-containing compounds, probably in form of SO_x_ gas. Functional groups containing oxygen such as hydroxyl, carboxyl and carbonyl on AC-DS (400 °C-KOH-acid) were more intense than that of raw DS powder due to the chemical activation process by KOH and acid-washing.

**Table 3:** Wavenumbers and possible functional groups for the peaks of FTIR spectra in Fig. 4f

#	Actual wavenumber (cm^−1^)	Range of wavenumber (cm^−1^)	Possible functional groups*
1	3305	3200–3400	O–H stretch for phenol and hydroxyl (strong & broad peak)
			N–H stretch for amide and 1″ and 2″ amine (medium peak)
2	2915	2800–3000	O–H stretch for carboxyl (variable & broad peak)
			C–H stretching for alkane (medium peak)
2	1735	~ 1700	C=O stretch for carbonyls, ester, aldehyde and ketone (strong peak)
4	1612	~ 1600	C=C stretch for alkene (medium peak)
5	1431	~ 1400	C–H bend and rock for alkane (medium peak)
6	1249	1200–1300	C–H wag for haloalkane (medium peak)
7	1081	1000–1100	C–O stretch for carboxyl and hydroxyl (variable peak)
8	1016	1040–1050	C–O stretch for carboxyl and hydroxyl (variable peak)
		1030–1070	S=O stretch for sulfoxide (strong peak)
		995–985	C=C bend for alkene (strong peak)
9	763	700–800	N–H wag for 1″ and 2″ amine (strong & broad peak)
			C–H bend for alkene (strong & broad peak)

**Fig. 5:**
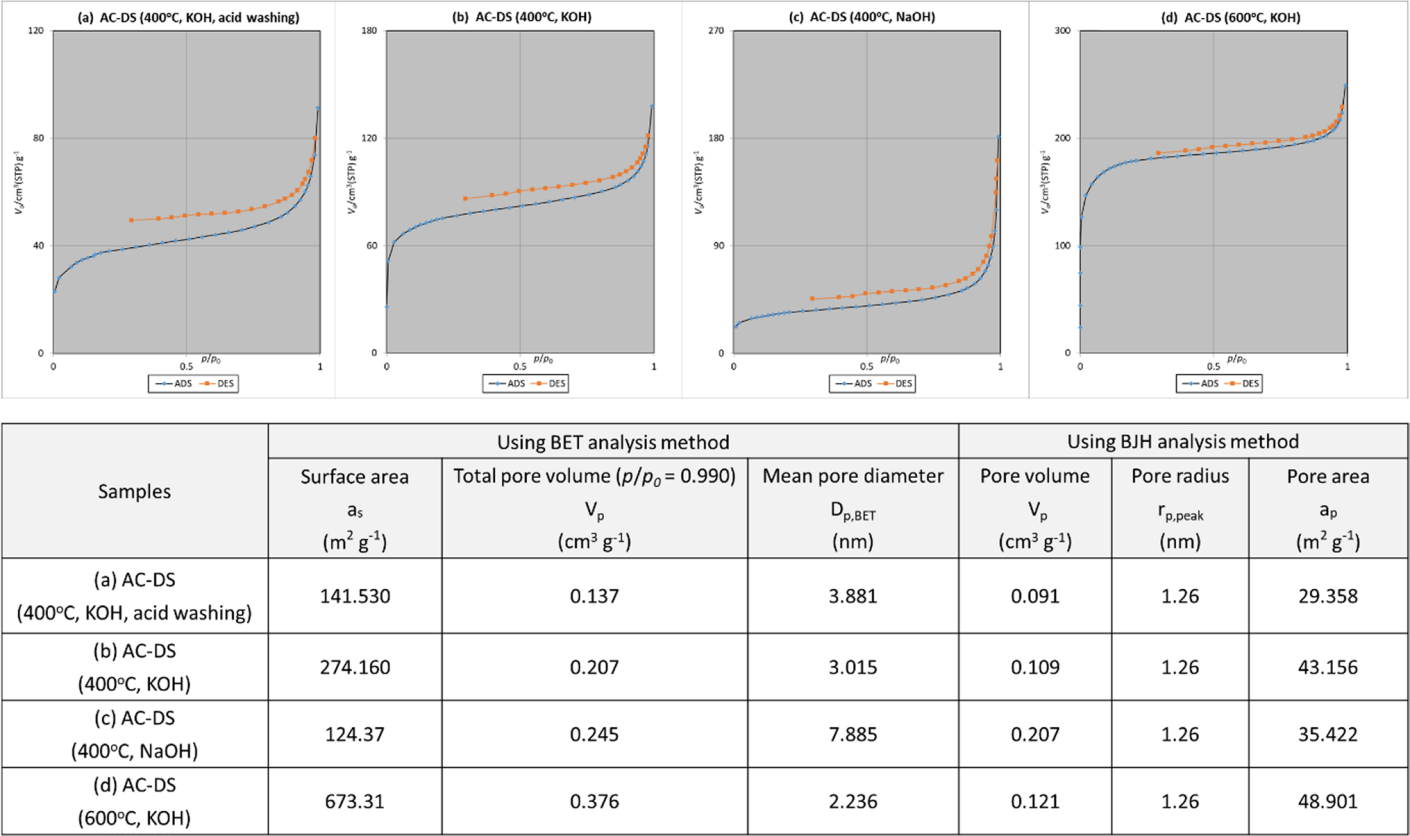
N_2_ adsorption and desorption isotherm plots for **a** AC-DS synthesised at 400 °C with KOH activating agent and with acid-washing, **b** AC-DS synthesised at 400 °C with KOH activating agent and without acid-washing, **c** AC-DS synthesised at 400 °C with NaOH activating agent and without acid-washing and **d** AC-DS synthesised at 600 °C with KOH activating agent and without acid-washing. Bottom table shows the characteristic of surface area and pores of AC-DS based on BET and BJH analysis methods

**Fig. 6:**
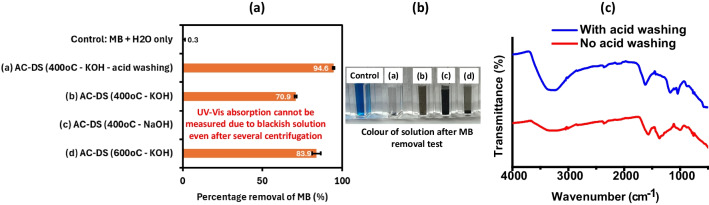
**a** Repeated experiment for the MB removal test of the new batch of synthesised AC-DS. **b** The image shows the colour of solutions after the MB removal test. The test was done with 0.5 mL of MB solution (0.125 g/L) and 0.5 mL deionized water and was left in the dark for 20 h. **c** FTIR spectra compare the effects of acid-washing to the AC-DS synthesised with KOH as activating agent and at 400 °C

The surface area and pore size distribution of the AC-DS were determined using N_2_ gas adsorption test with BET and BJH analysis methods. Based on the N_2_ gas adsorption and desorption isotherms, in Fig. 5a–d, all synthesised AC-DS at temperature of 400 °C closely resemble Type IV H3 hysteresis loop with a mesoporous structure [42]. Table at the bottom of Fig. 5 shows the surface area and pores characteristics of the AC-DS based on the BET and BJH analysis methods. The mesoporous structure of all AC-DS was also supported by the diameter of the pores from BET analysis which was within the 2 to 50 nm [43]. Although AC-DS synthesised with KOH activating agent, calcined at 400 °C and washed with acid showed the highest MB removal (Fig. 3), its BET surface area was the second lowest (141.53 m^2^ g^−1^) and BJH pore volume was the lowest among all AC-DS, indicating that the removal efficiency of the AC-DS was not mainly influenced by its surface area and pore volume. We further confirmed our results by repeating the MB removal test using a new batch of all AC-DS. In a repeated study (Fig. 6), we observed similar trend with our previous results in Fig. 3, except for this time, we were unable to get a proper UV–Vis absorption value for mixture of MB with AC-DS synthesised with NaOH as activating agent. Similarly, we observed dark brownish or blackish solution during the MB removal test when using AC-DS synthesised with NaOH as activating agent. We also compared the FTIR analyses of AC-DS samples with and without acid washing to further understand the influential factors to the performance of AC-DS in the MB removal. Based on the FTIR analysis in Fig. 6c, we found that the addition of acid washing increased the oxygen-containing functional groups (i.e., carboxyl and hydroxyl) to the AC-DS. Other than common high surface area and pore volume, the presence of these functional groups could be the main and more significant factor for the removal of MB in solution by AC-DS.

### Immobilisation of AgNPs on AC-DS

**Fig. 7:**
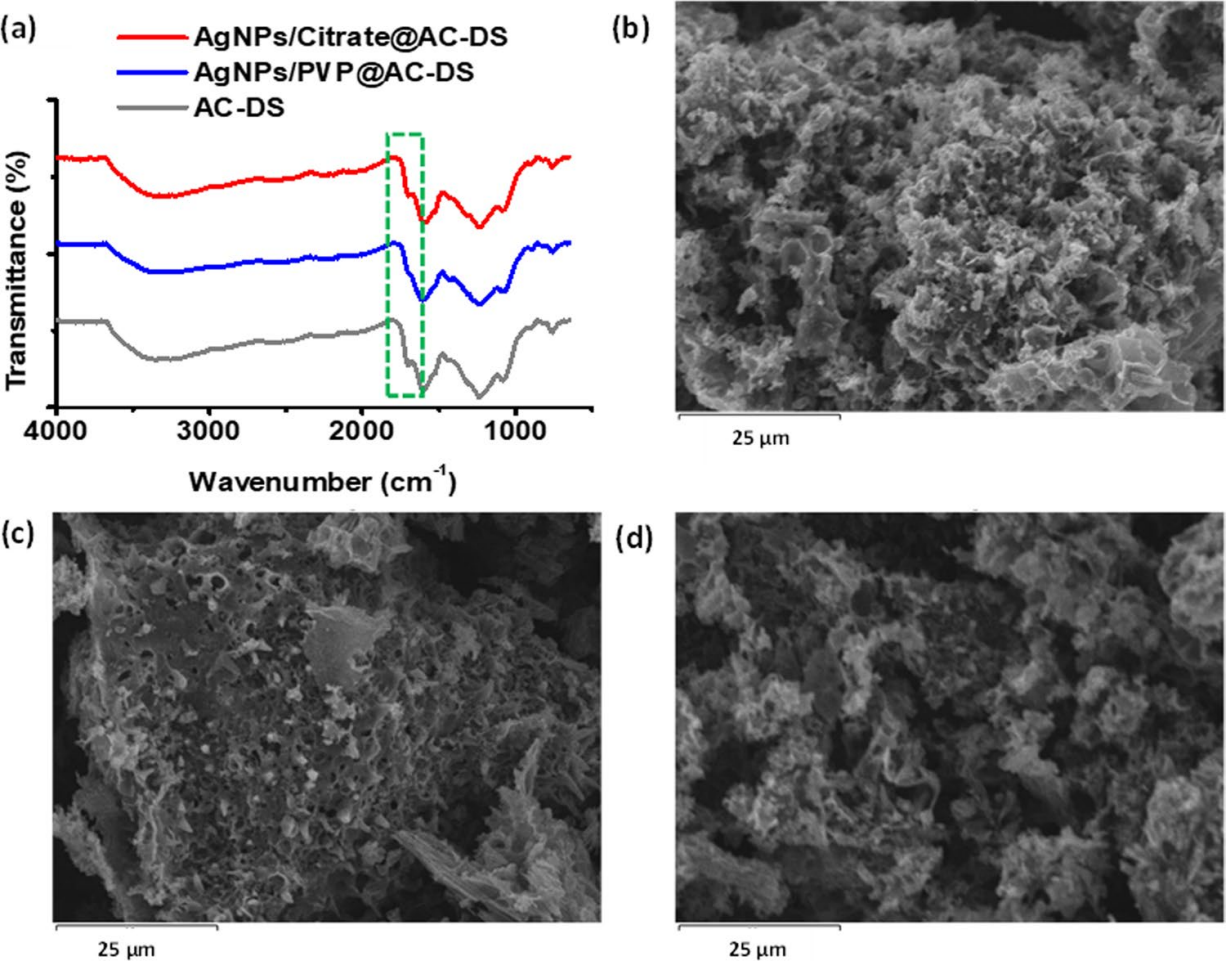
**a** Comparisons of FTIR spectra in Y-offset multiple graphs plot. From top to bottom: AgNPs/Citrate@AC-DS, AgNPs/PVP@AC-DS and AC-DS only. SEM images of **b** AC-DS only, **c** AgNPs/PVP@AC-DS, and **d** AgNPs/Citrate@AC-DS

**Fig. 8:**
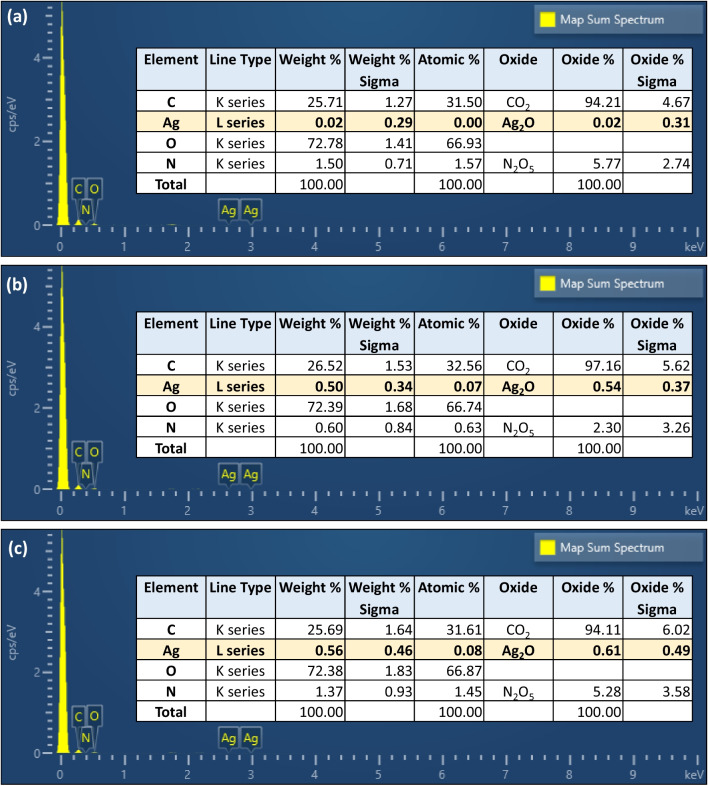
Elemental map spectrum analysis of **a** AC-DS only, **b** AgNPs/PVP@AC-DS and **c** AgNPs/Citrate@AC-DS by using SEM–EDS analysis

Results in Fig. 7 show the characterisations of AC-DS immobilised with AgNPs (abbreviated as AgNPs/PVP@AC-DS and AgNPs/Citrate@AC-DS). Based on the FTIR result in Fig. 7a, there was no distinctive difference in the surface functional groups of AC-DS alone and AC-DS immobilised with AgNPs. Small differences can only be seen in the region labelled by a green dashed rectangle where the peak belongs to the C=O stretching of carbonyls, aldehyde, and ketones. This peak is smaller and almost invisible for AgNPs/PVP@AC-DS as compared to AgNPs/Citrate@AC-DS, probably due to lack of C=O functional group in PVP as compared to citrate molecules (citrate molecule contains 3 carboxyl functional groups). Based on the SEM characterisation in Fig. 5b–d, it was hard to observe the presence of AgNPs on the surface of AC-DS, probably due to its low content. The low content of AgNPs on the AC-DS surface was further confirmed by the SEM–EDS analysis in Fig. 8a–c.

Based on the results of SEM–EDS analysis in Fig. 8a–c, it was found that AgNPs/Citrate@AC-DS had the highest AgNPs contents as compared to AgNPs/PVP@AC-DS which was indicated by the weight, atomic, and oxide percentages of Ag and AgO on the surface of AC-DS. In terms of weight percentage, AgNPs/Citrate@AC-DS, AgNPs/PVP@AC-DS, and AC-DS had 0.56%, 0.5%, and 0.02% respectively. In terms of atomic percentage, AgNPs/Citrate@AC-DS, AgNPs/PVP@AC-DS, and AC-DS had 0.08%, 0.07%, and 0% respectively. Meanwhile, in terms of oxide percentage, which is in the form of Ag_2_O, AgNPs/Citrate@AC-DS, AgNPs/PVP@AC-DS and AC-DS had 0.61%, 0.52%, and 0.02% respectively. All results show that the content of AgNPs on AC-DS was less than 1%, thus it was difficult to observe their presence under SEM analysis. In the synthesis of AgNPs and their immobilisation on AC-DS, stabilisers are required to act as a barrier, shielding the nanoparticles from environmental factors such as oxygen or moisture, which can lead to surface oxidation and loss of particle stability [44] which can further reduce the catalytic capability of the AgNPs. The stabilisers also act as a linker for the attachment between the AgNPs and the AC-DS surface.

**Fig. 9:**
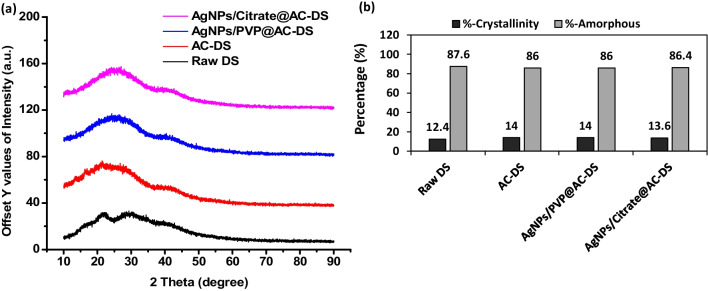
**a** Graph and **b** crystallinity and amorphous percentage of raw DS, AC-DS, AgNPs/PVP@AC-DS and AgNPs/Citrate@AC-DS from XRD analysis

XRD analysis was also done to the AC-DS and AC-DS immobilised with AgNPs. Based on existing studies, the common diffraction peaks for AgNPs can be observed at the 2$\theta$ values of 38.0°, 44.2°, 64.3°, 77.4° and 81.4° which can be assigned to the (111), (200), (220), (311), and (222) planes of the face-centered cubic (fcc) structure of Ag, matching with the data in the literature (JCPDS card number of 04-0783) [18, 45]. However, in our study, as shown in Fig. 9, the peak for silver cannot be seen probably due to the limitation of the XRD machine that we used and due to the little content of the AgNPs (about 0.5 wt% of Ag based on SEM–EDS in Fig. 8) in the AgNPs/Citrate@AC-DS and AgNPs/PVP@AC-DS. It was also found that the percentage of the crystallinity and the amorphous structure of the samples are quite comparable, also due to the extremely low content of the AgNPs immobilised on the AC-DS.

**Fig. 10:**
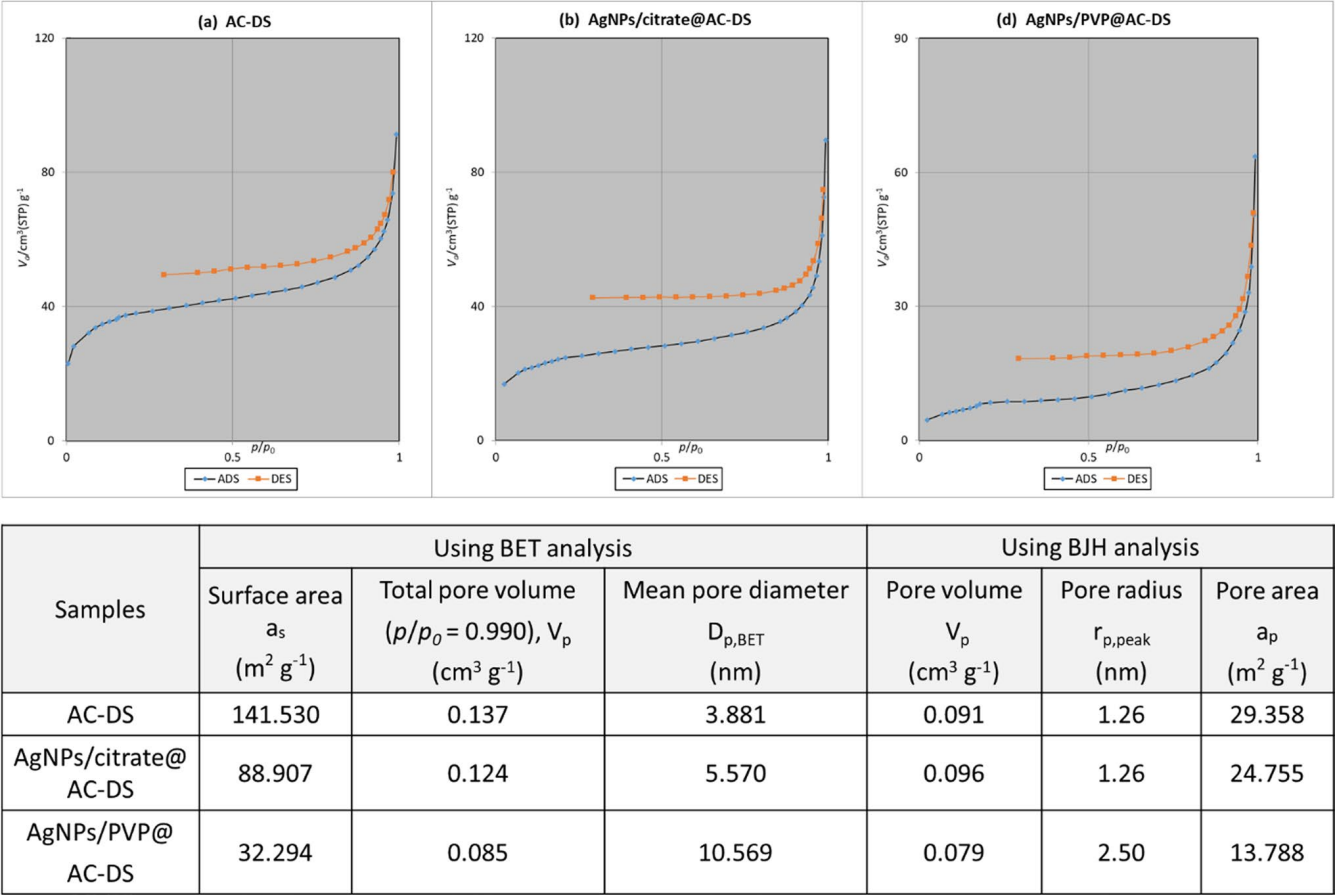
N_2_ adsorption and desorption isotherm plots for **a** selected AC-DS (synthesised at 400 °C with KOH activating agent and with acid-washing, **b** AgNPs/Citrate@AC-DS and **c** AgNPs/PVP@AC-DS. Bottom table shows the characteristic of surface area and pores of AC-DS based on BET and BJH analysis methods

Figure 10 shows the comparisons of N_2_ adsorption and desorption isotherm and BET and BJH analyses of selected AC-DS and its samples with immobilised AgNPs. Based on the graphs, it can be said that the addition of immobilised AgNPs did not cause much changes to the mesoporous structure of the AC-DS as they are all resemble Type IV H3 hysteresis loop with a mesoporous structure [42]. However, our results show that the addition of the AgNPs significantly reduced the BET surface area of the AC-DS. The AgNPs/PVP reduced almost 5 times of the BET surface area (from 141.53 m^2^ g^−1^ for AC-DS alone to 32.294 m^2^ g^−1^ for AgNPs/PVP@AC-DS), probably due to the usage of polymeric PVP which is a big and bulky molecule and could block the pores of the AC-DS. The BET total pore volume and BJH pore volume of AgNPs/PVP@AC-DS were also the lowest which could support our claim that the PVP might block the pores of the AC-DS. During the immobilisation of AgNPs onto AC-DS, we also found that some AgNPs/PVP couldn’t be immobilised on AC-DS as the colour of supernatant after centrifugation of AC-DS and AgNPs/PVP mixture was still brownish indicating some of the AgNPs/PVP were still in the supernatant. This observation was absent with AgNPs/Citrate and AC-DS mixture which resulted in almost clear supernatant solution after centrifugation, indicating that most of AgNPs/Citrate were immobilised on the AC-DS.

### Recyclability of AC-DS and AC-DS immobilised with AgNPs

**Fig. 11:**
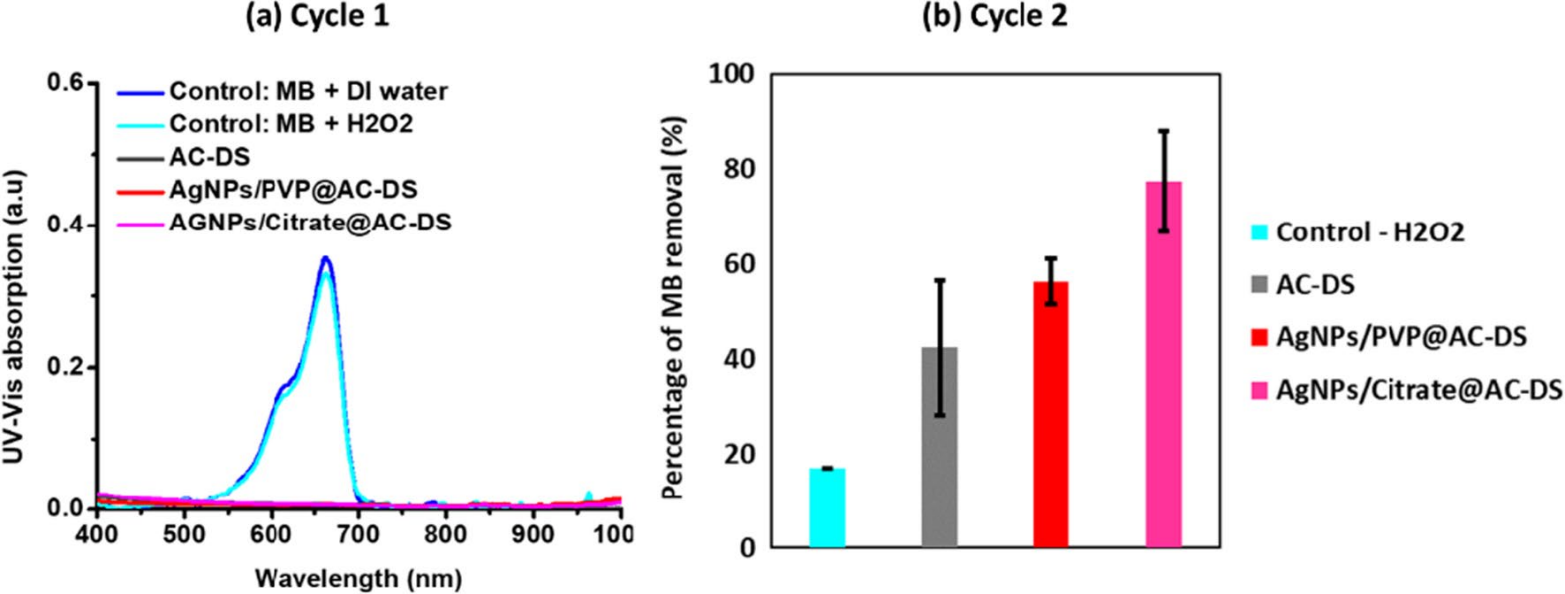
**a** UV–Vis absorption spectra of MB for MB removal by oxidation reaction in Cycle 1, and **b** Bar chat of percentage of MB removal by oxidation reaction in Cycle 2

The effects of immobilised AgNPs on AC-DS surface to the recyclability of AC-DS were determined by MB removal test, but with oxidation reaction which used H_2_O_2_ (20%) as the oxidizing agent. The results are shown in Fig. 11a, b. In the first cycle, a long duration (2 days) for the oxidation reaction was made to ensure full adsorption of MB occurred on the adsorption sites of AC-DS which was confirmed by the normalized plots in Fig. 11a. After that, the same MB removal test by oxidation reaction was repeated but the reaction time was reduced to an hour to ensure the differences of the MB adsorption together with its catalytic oxidation by the samples can be observed. From the bar chart in Fig. 11b, the AgNPs/Citrate@AC-DS resulted in the highest percentage of MB removal (77.44 ± 13%) as compared to AgNPs/PVP@AC-DS (56.19 ± 18%).

In addition, AC-DS immobilised with both AgNPs had better recyclability than the AC-DS alone (only 42.33 ± 33.34% of MB removal) showing about 50% improvement to the percentage of MB removal after being used once when the AC-DS was immobilised with AgNPs/Citrate, as compared to the AC-DS alone. These results indicate that the AgNPs that catalyzed the degradation of adsorbed MB by oxidation reaction had facilitated the regeneration of the adsorption sites on the AC-DS surface, thus improving its recyclability. Between PVP and citrate, it was found that citrate was a better stabiliser in the MB removal by oxidation reactions probably due to the presence of many carboxyl functional groups on a citrate molecule. The carboxyl groups which are always negative when in an aqueous solution due to deprotonation could increase the affinity of the MB molecule which contains positive functional groups (from sulfur and nitrogen) toward the catalytic site of the AgNPs, making the degradation of the MB by oxidation reaction happened more effective than that of AgNPs/PVP@AC-DS. On the other hand, PVP is a polymer that is bulky as compared to citrate, thus causing steric hindrance which reduces the accessibility of the MB to the catalytic sites of AgNPs.

**Fig. 12:**
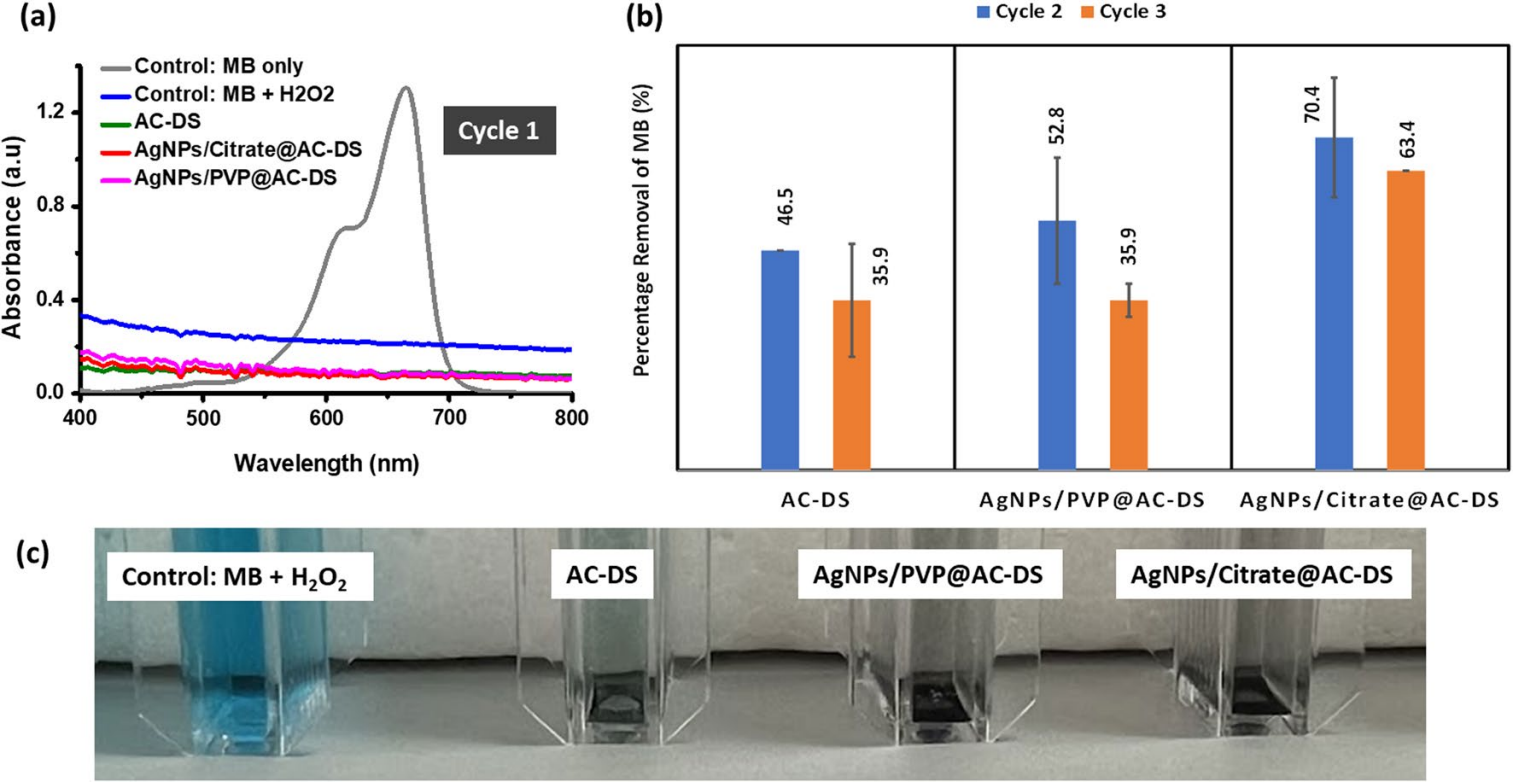
**a** UV–Vis absorption spectra of MB for MB removal by oxidation reaction in Cycle 1; **b** Bar chat of percentage of MB removal by oxidation reaction in Cycle 2 and 3; and **c** colour of MB solution with each sample after Cycle 3

The recyclability study was also repeated, and the cycles of the MB removal was increased. As shown in Fig. 12, we were only able to study the recyclability for up to 3 cycles only because there was a significant reduction in the amount of samples occurred during the transfer of the liquid to the cuvette after each cycle. Based on the results in Fig. 12a–c, we also found that the AgNPs/Citrate@AC-DS resulted in the highest percentage of MB removal as compared to AgNPs/PVP@AC-DS and AC-DS alone after recycling the sample twice (cycle 2 and cycle 3). Furthermore, both AC-DS with immobilised AgNPs demonstrated superior percentage of MB removal and exhibited enhanced recyclability compared to AC-DS alone. Notably, the blue colour of MB persisted in the solution of AC-DS, indicating low recyclability of AC-DS alone in the MB removal process.

**Fig. 13:**
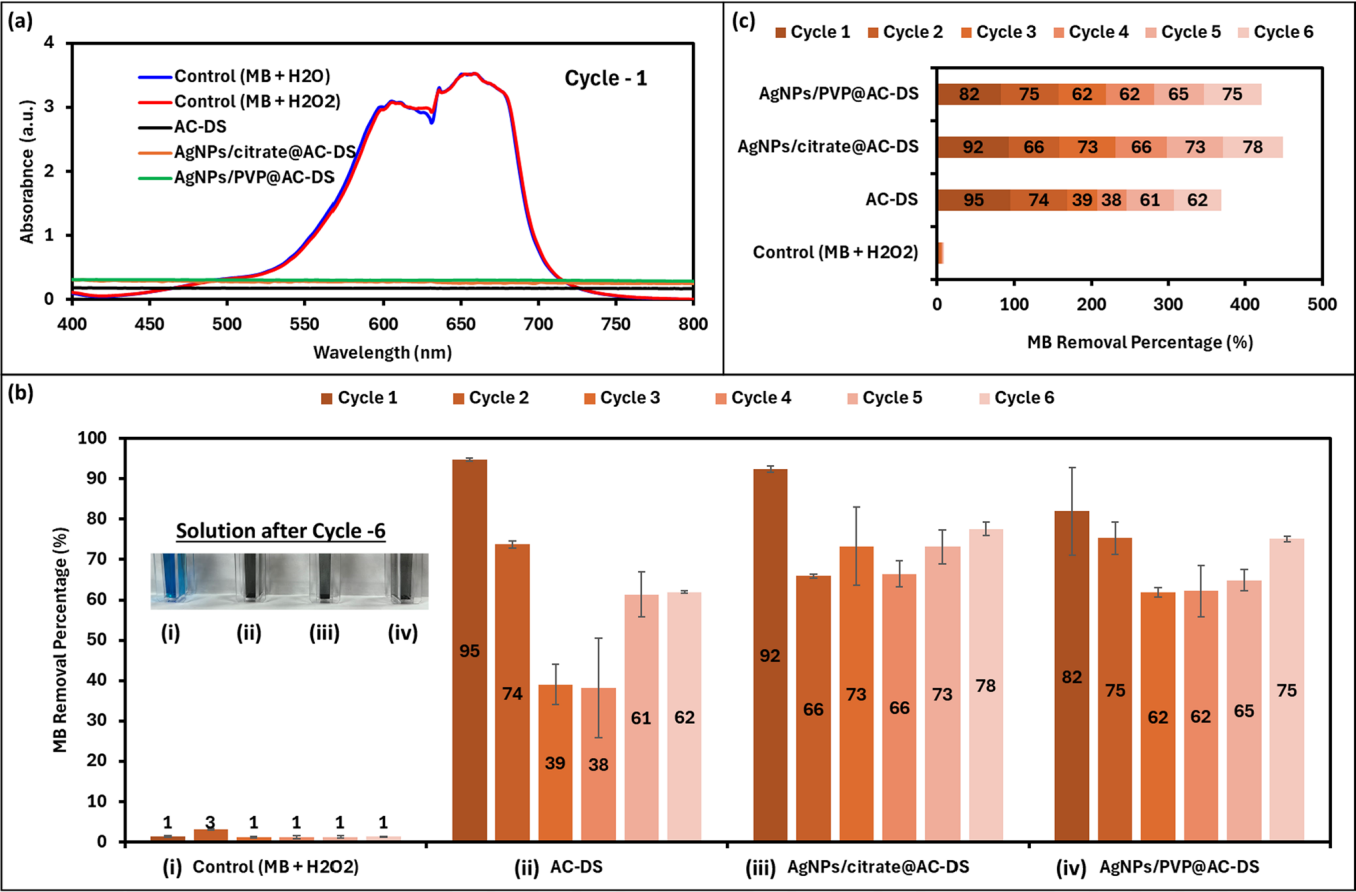
**a** UV–Vis absorption spectra of MB for MB removal by oxidation reaction in Cycle 1; **b** Vertical bar chat of percentage of MB removal in Cycle 2 to Cycle 6 with image of solutions after Cycle 6 and **c** Horizontal bar chat of  overall percentage of MB removal from Cycle 1 to Cycle 6

Third recyclability test was also performed, and the experiment was done for up to 6 cycles, using a modified method to prevent the reduction of AC-DS samples for the next cycles. This experiment was also done using a new batch of the samples. Based on the results in Fig. 13a–c, in overall, it can be said that AgNPs/Citrate@AC-DS resulted in the highest of MB removal and the best recyclability, followed by AgNPs/PVP@AC-DS and AC-DS alone. Although the surface area and the pore volume of the AgNPs/Citrate@AC-DS were lower than that of AC-DS alone, the addition of AgNPs/Citrate caused higher MB removal probably due to higher carboxyl and hydroxyl functional groups, which were contributed by the citrate molecules, the stabilisers used in the synthesis of AgNPs/Citrate. The findings also indicate the more significant effects of functional groups than the surface area and pore structure of AC-DS in the MB removal by adsorption and catalytic oxidation.

Notably, we have tried to identify and differentiate the percentage of MB removal by adsorption on AC-DS and catalytic oxidation of MB by the AgNPs immobilised on the AC-DS, however, we have encountered challenges, probably due to the small size and composition (less than 1%) of AgNPs. Our observations suggest that the rapid removal of MB through adsorption onto the AC surface can outpace the catalytic oxidative removal of MB by AgNPs immobilised on the AC-DS. Consequently, it becomes intricate to distinctly attribute the removal to either adsorption or catalytic activities in a single test. In light of these complexities, we have focused on presenting the results of the recyclability of AgNPs/Citrate@AC-DS and AgNPs/PVP@AC-DS in comparison to AC-DS alone. This approach provides indirect evidence regarding the catalytic activity of AgNPs in MB removal.

As depicted in recyclability tests, the MB removal of AC-DS incorporated with AgNPs was higher than that of AC-DS alone in the second cycle (Figs. 11, 12) and after the third cycle (Fig. 13). The increase in MB removal during the second cycle and the third cycle indirectly suggests that, after the initial adsorption of MB by AC-DS in the first cycle, the presence of AgNPs in AgNPs/Citrate@AC-DS and AgNPs/PVP@AC-DS catalytically oxidizes the MB, leading to its degradation into smaller molecules. This catalytic process enables the products of degraded MB molecules to leave the AC-DS surface, thereby freeing up more adsorption sites on AgNPs/Citrate@AC-DS and AgNPs/PVP@AC-DS for subsequent cycles.

### Leaching of AgNPs from AC-DS

This study was performed to determine the leaching of AgNPs from AC-DS. As we added H_2_O_2_, a quite strong oxidants in the MB removal test, there might be oxidation reaction happened to the AgNPs, forming more Ag^+1^ ions inside the mixture. Due to the absent of inductively coupled plasma—optical emission spectrometry (ICP-OES) machine in our university and limited access to the machine elsewhere, we designed a simple experiment to provide indirect evidence on the leaching of AgNPs. After the MB test, we collected the supernatant from the centrifuged mixture, and we add 5 mm filter paper inside the supernatant and let the supernatant dry in the oven at 60 °C. It was hypothesized that any AgNPs or Ag^+1^ ions will be attached to the filter paper when drying. The filter paper was then characterised under SEM–EDS to determine the weight and atomic percentage of silver and oxygen atoms. The weight and atomic percentages of silver and oxygen from SEM–EDS analysis were tabulated in Table 4. As can be seen from the results, the leaching of AgNPs did occur after the first cycle of the MB removal test. However, its amount is considerably low as compared to the control AgNPs solution only. When comparing with the silver weight percentage of AgNPs solution, it was about 10.7% of AgNPs/Citrate leached out from the AgNPs/Citrate@AC-DS after the first cycle of the MB removal. Meanwhile, it was about 25% of AgNPs/PVPs leached out from the AgNPs/PVP@AC-DS after the first cycle of the MB removal. Further study can be done to fully understand the strength and dynamic attachment of AgNPs on the AC for improving the capability of the metal nanoparticles in enhancing the performance and recyclability of AC.

**Table 4:** Weight and atomic percentages of silver and oxygen by SEM–EDS. The magnification of the sample was 200k with operating conditions of 20 kV and working distance of 10 mm

Samples	Silver content		Oxygen content	
	Weight%	Atomic%	Weight%	Atomic%
Control 1: AgNPs/Citrate solution	2.8 ± 0.6	0.35 ± 0.1	99.1 ± 1.6	99.9 ± 0.2
Control2: AgNPs/PVP solution	2.0 ± 0.0	0.40 ± 0.1	99.3 ± 1.2	99.9 ± 0.2
Control 1: AC-DS	0.0 ± 0.0	0.0 ± 0.0	99.7 ± 0.5	100.0 ± 0.0
AgNPs/Citrate@AC-DS	0.3 ± 0.5	0.0 ± 0.0	99.7 ± 0.5	100.0 ± 0.0
AgNPs/PVP@AC-DS	0.5 ± 0.4	0.1 ± 0.0	99.8 ± 0.2	99.9 ± 0.0

As previously stated, to the best of our knowledge and through a comprehensive comparison with existing studies (summarized in Table 5), our investigation stands out as it employs a combination of durian skin for AC synthesis, coupled with the immobilisation of metal nanocatalysts, which were AgNPs to enhance the recyclability or reusability of the AC-DS. The in-house synthesis of AgNPs employed distinct stabilisers, namely polyvinylpyrrolidone polymer and anionic citrate molecules. Notably, this study uniquely explores the comparative efficacy of various stabilisers, highlighting the significant influence of the oxygen containing functional groups which were contributed by acid washing and citrate stabilisers and emphasizes the recyclability of the AC derived from durian skin in the removal of methylene blue from solution. Consequently, this research contributes valuable insights into the role of metal nanoparticles and stabiliser types, thereby elucidating their impact on the recyclability and adsorption properties of AC.

**Table 5:** Comparisons of methodology and findings in this study with other existing studies

No	References	Type of adsorbent	Metal nanoparticles (MNPs)	Removal of MB	Findings
1	This study	Activated carbon synthesised from durian skin (AC-DS) Study the effects of reaction conditions	AgNPs were synthesised with stabilisers (polyvinylpyrrolidone and citrate) Comparing the effects of different types of stabilisers Content of Ag was less 0.5% (less than 1%) based on SEM–EDS	Used 10 mg of AC-DS with and without AgNPs 1.5 mL of 0.2 g/L of MB used for adsorption test of AC-DS 0.5 mL of 0.125 g/L of MB used for recyclability test Catalytic oxidation reaction using H_2_O_2_ was used for catalytic degradation of MB	More than 90% removal of MB by the AC-DS About 50% improvement to recyclability of AC-DS when incorporated with AgNPs/Citrate (77% removal of MB after second cycle)
2	[46]	Commercial granular AC purchased from Tra Bac Company	AgNPs synthesised with starch as stabiliser 0.65 wt % of Ag based on EDS	MB (25 mL; 100, 500 and 1000 mg/L) and AgNPs-AC (50, 250, 500 mg) used for the adsorption test Adsorption isotherm was studied	Achieved up to 90% removal of MB
3	[47]	AC from coconut shell	AgNPs and TiONPs 11.6 wt % of Ag in the nanocomposite	MB (10–50 mg/L) and CSAC@AgNPs@TiO2NPs (50 mg) used for adsorption test Effects of adsorption parameters and adsorption kinetics were studied Recyclability/reusability was studied	More than 85% removal of MB after 6 cycles
4	[48]	AC synthesised from residue-coconut husk	AgNPs synthesised by sono-electrochemical method Average size of AgNPs was 23 nm	MB (100–500 mg/L; 200 mL) and AgAC (0.2 g) used for adsorption test	70% removal of MB
5	[42]	AC synthesised from cashew nutshell (CNSAC)	AgNPs synthesised using in-situ reduction on the CNSAC Content of Ag in the nanocomposite was 3.04 wt % based on EDX	MB (100 mL; 50 mg/L) and catalysts (0.2 g/L)	Removal of MB was 98.3% after 120 min
6	[15]	Commercial AC purchased from Merck	Comparing AgNPs and PdNPs The AgNPs and PdNPs were synthesised by in situ reduction method without the addition of stabilisers/ligands Size of AgNPs (15 nm–80 nm) Content (wt or atomic %) of Ag on AC was unknown	Study the effect of parameters (pH, adsorbent weight, contact time, initial dye concentration) in the adsorption of MB Study the adsorption isotherm and kinetics	Adsorption of MB onto AgNPs-AC and PdNPs-AC follows Langmuir model and second order kinetic model About 95% removal of MB by AgNPs-AC
7	[18]	Polymer-based adsorbent (Polystyrene-divinylbenzene mesoporous microsphere)	AgNPs synthesised by in-situ reduction method without the addition of stabilisers/ligands Content of AgNPs was about 25.48 wt % by thermogravimetric analyses	1 mg of micropsheres and 2 mL of 0.15 mM MB used for adsorption test Catalytic reduction reaction using NaBH_4_ was used for catalytic removal of MB	Efficiency of 97.12% removal of MB even after 5 cycles of adsorption test
8	[19]	Silica (SG)	AgNPs, synthesised by in-situ reduction with Cicer arietinum pod extract as biogenic Effect of light to the catalytic activity of AgNPs-silica (Ag@SG) was studied AgNPs with spherical structure with size ranging from 6 – 14 nm AgNPs content on SG was unknown	50 mg Ag@SG and MB (50 ppm; 20 mL) was used for dark adsorption test 50 mg Ag@SG and MB (40 ppm; 50 mL) was used for photo adsorbent test Catalysis reaction without addition of oxidant or reductant Amount of catalysts, MB concentration and time were studied for the adsorption test	88% removal of MB by photodegradation by Ag@SG as compared to AgNPs and SG alone
9	[9]	Periwinkle shelled-hyroxyapatite (HAP)	AgNPs synthesised with Terminalia cattappa capping agents Rod-shape of AgNPs Content of AgNPs was about 7.7% (unknown basis of percentage)	AgNPs-HAP (0.5 g) and MB and Congo red (50 mL; 50 mg/L) used for adsorption test Influences of adsorption parameters and adsorption isotherm were studied Reusability/recyclability was also studied	Over 80% of percentage of dye removal at the third cycle

We also found other studies that used AgNPs immobilised on AC but mainly for capability other than as catalysts, which is as antibacterial agent [33, 42]. In another study, AgNPs immobilised on AC was used to remove methyl orange and it was found that AgNPs-coated AC was successfully recycled for 10 successive adsorption–desorption cycles indicating its high reusability [31].

## Conclusions

In conclusion, the conversion of waste into value-added products with improved performance is highly encouraging for sustainability. As in this study, AC was successfully synthesised from DS, a solid waste that is commonly discarded in landfills. It was found that 400 °C (as compared to 600 °C), KOH (as compared to NaOH), and the presence of acid-washing (50% of HNO_3_) resulted in AC with the highest removal of MB (91.49 ± 2.86%). In addition, the recyclability of the AC-DS in the removal of MB was improved by the immobilisation of metal nanocatalysts, AgNPs on the AC-DS surface. This study also shows that the type of stabiliser of AgNPs has profound effects on its immobilisation on AC-DS, its catalytic activity and recyclability of the AC-DS as the recyclability based on the percentage of MB removal in the second cycle (77.44 ± 13% and 56.19 ± 18% for AgNPs/Citrate@AC-DS and AgNPs/PVP@AC-DS, respectively) as compared to AC-DS alone (only 42.33 ± 33.34%). This study also highlights the significant effect of carboxyl and hydroxyl functional groups from the acid washing and from the stabiliser (citrate molecules) for the removal of MB as compared to surface area and pore structure of the AC-DS. These functional groups influence the accessibility of the catalytic sites of AgNPs for the catalysis, adsorption sites of AC-DS for adsorption and in making sure the attachment of AgNPs is strong enough to prevent them from leaching out from the surface of AC-DS during the MB removal test.

## Data Availability

The authors declare that the data supporting the findings of this study are available within the paper. Additional data related to our earlier study on the synthesis of silver nanoparticles (i.e., the UV–Vis absorption of silver nanoparticles solutions synthesised by different amount of stabilisers) have been published and available in the IOP Conference Series: Materials Science and Engineering (with the identifier 10.1088/1757-899X/1192/1/012031) and they may be used under the terms of the Creative Commons Attribution 3.0 license. Should any raw data files be needed in another format they are available from the corresponding author upon reasonable request. Source data are provided with this paper.

## Abbreviations

AC: Activated carbon
AgNPs: Silver nanoparticles
DS: Durian skin
MB: Methylene blue
PVP: Polyvinylpyrrolidone
